# Recent advances in understanding cell type transitions during dorsal neural tube development

**DOI:** 10.12703/r/11-27

**Published:** 2022-09-27

**Authors:** Chaya Kalcheim, Dina Rekler

**Affiliations:** 1 Department of Medical Neurobiology, Institute of Medical Research Israel-Canada (IMRIC) and the Edmond and Lily Safra Center for Brain Sciences (ELSC), Hebrew University of Jerusalem-Hadassah Medical School, Jerusalem, Israel

**Keywords:** BMP, cell cycle, definitive roof plate, dorsal interneurons, ependymal cells, epithelial to mesenchymal transition, neural crest, neural tube, radial glia, retinoic acid, somite, Wnt

## Abstract

The vertebrate neural tube is a representative example of a morphogen-patterned tissue that generates different cell types with spatial and temporal precision. More specifically, the development of the dorsal region of the neural tube is of particular interest because of its highly dynamic behavior. First, early premigratory neural crest progenitors undergo an epithelial-to-mesenchymal transition, exit the neural primordium, and generate, among many derivatives, most of the peripheral nervous system. Subsequently, the dorsal neural tube becomes populated by definitive roof plate cells that constitute an organizing center for dorsal interneurons and guide axonal patterning. In turn, roof plate cells transform into dorsal radial glia that contributes to and shapes the formation of the dorsal ependyma of the central nervous system.

To form a normal functional spinal cord, these extraordinary transitions should be tightly regulated in time and space. Thus far, the underlying cellular changes and molecular mechanisms are only beginning to be uncovered. In this review, we discuss recent results that shed light on the end of neural crest production and delamination, the early formation of the definitive roof plate, and its further maturation into radial glia. The last of these processes culminate in the formation of the dorsal ependyma, a component of the stem cell niche of the central nervous system. We highlight how similar mechanisms operate throughout these transitions, which may serve to reveal common design principles applicable to the ontogeny of epithelial tissues.

## Introduction

During embryonic development, dynamic cellular events are decisive for the appropriate organization of the definitive body plan, proper cellular differentiation, and eventual tissue and organ function. A representative example of an embryonic domain exhibiting continuous changes over time is provided by the development of the dorsal domain of the neural tube (NT). This region first becomes apparent during NT closure, when bilateral neural folds come close to each other and fuse in the midline^1–4^. Opposing morphogen gradients generate distinct identities along the dorso-ventral axis of the NT. Bone morphogenetic protein (BMP) and Wnt emanate from the dorsal NT and act across the dorsal third of the NT^5,6^, Sonic hedgehog produced in notochord and later floor plate acts primarily in the ventral region, and retinoic acid (RA) from the paraxial mesoderm acts at an early stage to pattern the dorso-ventral and rostro-caudal embryonic axes^7–10^. The integration of the above signals establishes a transcriptional gene regulatory network that first defines the premigratory neural crest (NC)^11,12^, an epithelial cell subset that transiently populates the NT. Owing to a remarkable process of epithelial-to-mesenchymal transition (EMT), these progenitors leave the NT^13^ to form a rich collection of cell types, such as sensory and autonomic neurons, satellite cells, and Schwann cells of the peripheral nervous system as well as pigment cells, ectomesenchyme and endocrine derivatives, organized in combinations unique to specific axial levels^14,15^. Upon completion of NC production and emigration, a subset of progenitors initially localized ventral to the premigratory cohort of NC cells, relocate dorsally to home at the dorsal NT midline, where they become the definitive roof plate (RP) of the spinal cord^16^. The RP is flanked ventrally by dorsal interneurons and significantly contributes to the specification and/or differentiation of selected interneuron populations^17–20^. Subsequently, the RP is transformed into radial glia (RG)-like cells^21–24^ that shape the growth of spinal cord axons and contribute to generating the stem cell-containing dorsal ependymal zone of the adult spinal cord of humans and rodents^21,25,26^.

A key question imposed by the above knowledge is the elucidation of temporal mechanisms. For example, how does the sequential production of different cell types at the same location, but not necessarily from the same progenitors, contribute to the generation of cell type diversity? In spite of extensive knowledge on the formation, emigration, and migration of NC progenitors, little is known about the mechanisms that account for the completion of the NC period. Furthermore, how is the ensuing formation of the RP regulated? Are the end of NC and the establishment of the RP interconnected or autonomously regulated processes? What mechanisms account for the transformation of epithelial RP cells into RG during the transition into a mature spinal cord structure? How do RG cells affect the formation of the local ependyma? Recent studies, as discussed here, have begun clarifying meaningful cellular and molecular processes underlying these important events and set the basis for further in-depth investigation.

## The end of neural crest production and emigration

The end of NC production entails a set of significant changes occurring in the dorsal NT. These include the downregulation of NC-specific genes, a structural reorganization of the region, and the cessation of EMT and cell emigration. Our understanding of the end of NC production and the transition into an RP was hindered by the lack of genes uniquely transcribed in either NC or RP populations, respectively. A recent transcriptome analysis, performed at the trunk level of quail embryos, compared the dorsal NT at premigratory NC and RP stages, respectively. This yielded a selection of genes expressed in RP but not premigratory NC, including components of the BMP, Wnt, and RA pathways, that serve both for defining these structures and for functional analysis, as discussed below in this section^27^. Initially, prospective NC and RP progenitors, being part of the FoxD3^+^ lineage, exhibit the same gene expression profile^28^. It is during a ventro-dorsal cellular relocation induced by the onset and progression of NC EMT that RP progenitors downregulate the NC genes *foxd3*, *snai2*, *sox9*, and so on, thus segregating from the NC lineage^28^. This is of particular interest as it implies that the end of cell emigration is not necessarily accounted for by the exhaustion of the NC progenitor pool but rather by a tightly regulated molecular switch.

Consistent with this notion, BMP signaling was shown to be necessary for the early development of both NC and RP populations^17,29–31^. This effect is transient, as at later stages dorsal progenitors lose competence to generate these cell types and instead yield dorsal interneurons^32^. Indeed, whereas both NC and RP progenitors exhibit BMP activity, the nascent RP becomes refractory to BMP^30^.

In a recent study, the loss of responsiveness to BMP was found to depend on RA newly produced in the dorsal NT^5^. Upon inhibition of RA signaling, expression of an NC-specific molecular signature, cell proliferation, lack of an organized apicobasal architecture, and (most importantly) cell emigration were all extended well into the RP stage^5^. These data show that the local synthesis of RA in the nascent RP, acting via BMP, is the switch that turns off the NC period of dorsal NT development.

**Figure 1.**
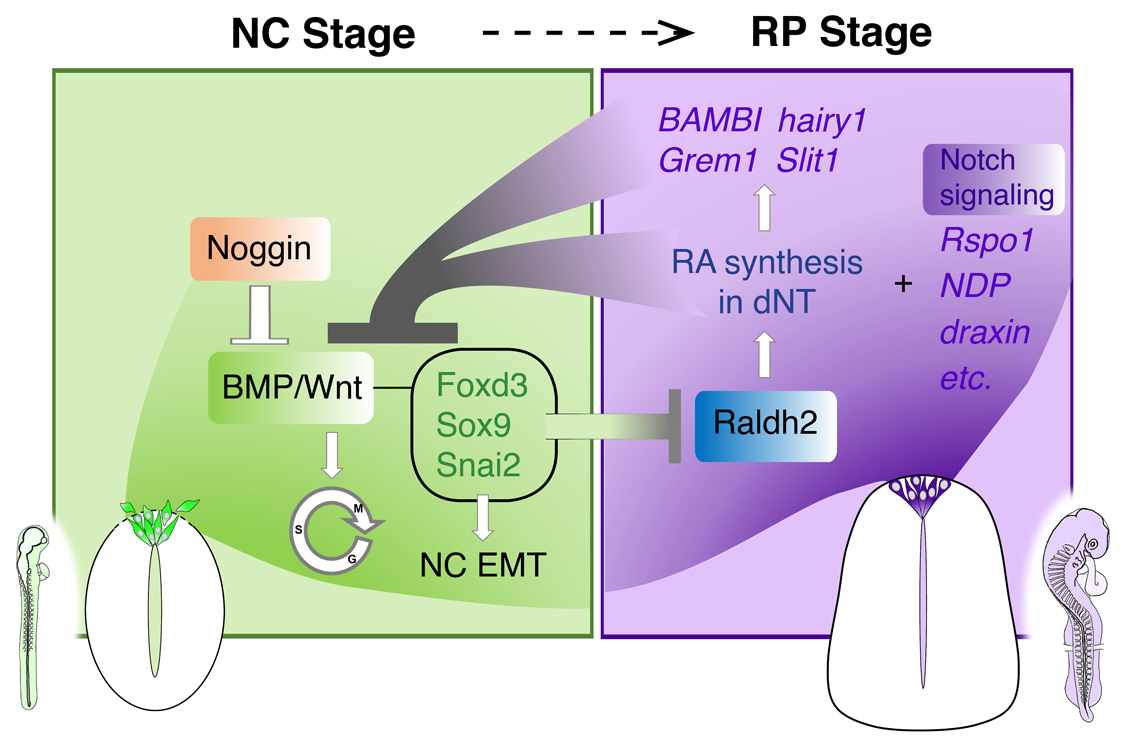
The role of retinoic acid (RA) in the transition from neural crest (NC) to roof plate (RP). A proposed model for the transition between NC (green) and RP (purple) stages. In the early stages, reciprocal gradients of RA and fibroblast growth factor in the paraxial mesoderm result in the downregulation of the bone morphogenetic protein (BMP) inhibitor Noggin in the dorsal neural tube (dNT). This leads to the activation of BMP and Wnt signaling pathways, which promote cell proliferation and induce NC epithelial-to-mesenchymal transition (EMT). As NC cells delaminate and leave the neural tube, NC-specific genes (*foxd3*, *sox9*, and *snai2*) are downregulated. The latter genes were shown to inhibit the synthesis of *Raldh2* in the nascent RP; thus, their disappearance enables the onset of *Raldh2* expression leading to the local synthesis of RA in RP. RA in turn inhibits BMP and consequently Wnt signaling, either directly or via upregulation of BMP inhibitors (*BAMBI*, *hairy1*, and *Grem1*). Hence, mutual cross-inhibitory interactions between NC- and RP-specific genes underlie the establishment of a temporal sequence leading to the formation of the definitive RP and its segregation from NC. In addition, Notch signaling stemming from the RP/interneuron boundary is essential for RP formation yet has no apparent effect on early NC development.

These results raised the obvious question of the mechanism underlying the onset of RA production in RP. The expression of Raldh2, the enzyme responsible for RA synthesis, is upregulated in RP concomitant with the downregulation of *foxd3*, *snai2*, and *sox9*. Furthermore, NC and RP genes stand in a mutually repressive interaction, suggesting that cross inhibition is a factor responsible for setting the timing of events in this domain^5^ (Figure 1).

## The transition into a definitive roof plate

In the previous section, we described that RA signaling emanating from the nascent RP itself inhibits BMP activity, which altogether accounts for the end of the NC stage^5^. A fundamental question is whether ending the production of NC cells is sufficient to induce RP formation or, alternatively, whether additional factors are required. Whereas inhibiting RA signaling was sufficient to prevent the timely expression of RP-specific genes that encode BMP inhibitors such as *BAMBI*, *Grem1*, and *hes4*, additional genes unique to RP, such as *Rspo1*, *draxin*, and *NDP*, were normally expressed^5^. This suggests that RP formation takes place under these conditions and thus can be mechanistically distinguished from its NC predecessors in the dorsal NT. However, the resulting RP was not normal, as the entire cytoarchitecture of the RP, comprising apicobasal polarity and lack of cell proliferation, was compromised^5^. Furthermore, single cells co-expressing both NC and RP traits were apparent. Together, this indicates that proper NC/RP lineage segregation requires RA-dependent termination of BMP signaling and typical BMP-dependent traits (Figure 1). This finding is consistent with, and provides further mechanistic insights on, recent data emphasizing that lineage determination in the NC system is initiated by co-activation of competing modules, followed by a cell fate bias before final segregation^33^. It would be relevant to examine whether the same molecular modules operate to control NC-to-RP transition in additional animal species.

The Dreher mutant mouse, a spontaneous neurological mutation defective in *Lmx1a*^34^, was also instrumental in addressing the potential role of BMP signaling in RP formation. Normally, *Lmx1a* expression is apparent in both premigratory NC and RP. In Dreher mutants, RP formation is perturbed with reduced formation of dI1 interneurons. Expression of *Lmx1a* was not maintained through the mature RP, yet it was unaltered in NC, which revealed no phenotypic changes. Notably, loss of *Lmx1a* was accompanied by a complete failure of *bmp6* and *gdf7* expression throughout NT development^35^, yet some features of the RP were still maintained in the trunk of the mutants. In the hindbrain, only certain rostro-caudal regions of the RP were lost^34,36^, indicating heterogeneity within the RP^27,37^ and/or that *Lmx1a-*dependent BMP signaling may not be necessary for all aspects of RP development.

Notch-Delta signaling was found to mediate the maintenance of the hindbrain RP epithelium^37^. Subsequent results documented a crucial role for this pathway in *de novo* RP formation. In the absence of Notch function, no RP or dI1 interneurons formed in mouse embryos. Reciprocally, the gain of Notch produced an ectopic RP at the RP-interneuron interface. Moreover, Notch signaling was found to be sufficient for the choice between RP and dI1 interneuron fates while exhibiting no effect on early NC development^27^. Notably, monitoring activated Notch revealed activity throughout the NT except within the RP, and so was the expression pattern of Notch ligands. Hence, Notch signaling drives the initiation of RP specification in neighboring cells, likely by establishing a boundary between prospective RP and dorsal interneurons^27^. Together, the precedent data lend experimental support to the idea that despite being sequentially produced, separate signals are needed for ending NC production and for stimulating the emergence of an RP.

## Sequential maturation of the roof plate into radial glia and dorsal ependyma

### Cell-cell interactions in remodeling of the dorsal neuroepithelium

**Figure 2.**
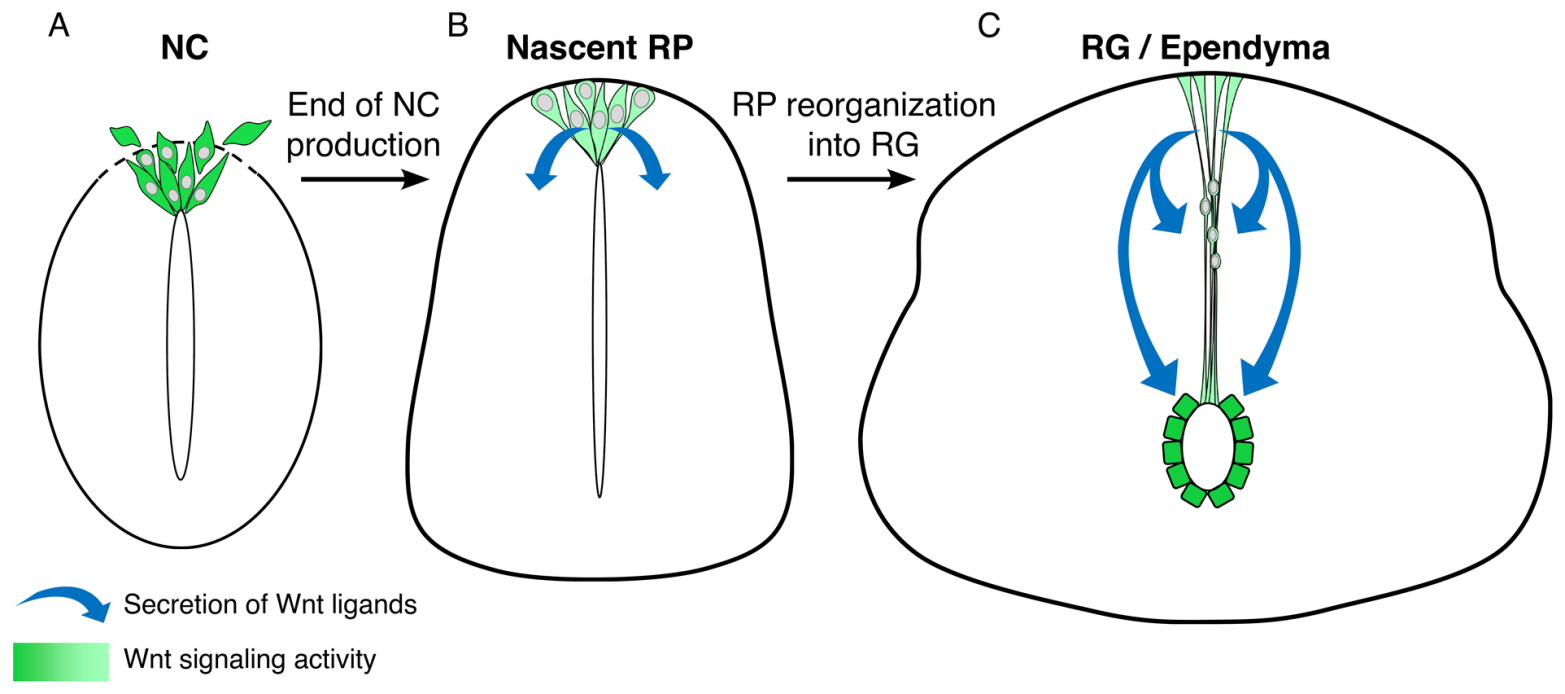
Reiterative roles of Wnt signaling throughout the maturation of the dorsal neural tube (NT). The dorsal NT undergoes a series of structural transformations during embryonic development. An initial neural crest (NC)-producing domain (**A**) turns into a definitive epithelial roof plate (RP) (**B**), which later reorganizes into radial glial (RG) cells stretched along the dorsal extent of the spinal cord between the central canal and the pia mater (**C**). (**A**) The dorsal NT continuously produces and secretes Wnt ligands (e.g., Wnt1 and Wnt3a) and also responds to Wnt signals. Premigratory NC cells exhibit high levels of Wnt signaling, responsible for their proliferation as well as for epithelial-to-mesenchymal transition. (**B**) During the transition into a definitive RP, Wnt activity persists at lower levels and the RP continues secreting Wnt ligands, important for the development of dorsal interneuron progenitors. (**C**) In mice, Wnt signaling in nascent RG was found to be necessary for their proper alignment in the dorsal midline (upper arrows). Later in development, Wnt ligands secreted from the ventral tips of dorsal RG are, in turn, crucial for the proliferation of dorsal ependymal cells (lower arrows).

During the transition from an NT composed of pseudostratified epithelial progenitors to a nascent spinal cord, emigrating NC progenitors are replaced by RP cells^16,28,30,38^. Subsequently, cellular morphology changes dramatically: the NT lumen gradually shrinks, forming the central canal, and a median septum extends along the dorso-ventral axis, a process that concerns the dorsal domain of the NT^21,23,24,39,40^ exclusively. Recent studies in zebrafish, chick, and mouse embryos revealed that the RP stretches during this transition with long processes abutting the central canal apically and the pial surface basally^26,39,41^ (Figure 2). These findings raised the questions of how precisely the NT lumen shrinks into a small and round central canal, and what the role of the elongating dorsal RG in this process is^23,24^.

A recent study addressed these questions. The authors describe the formation of a cellular bridge spanning the two sides of the dorsal ventricular lumen, located 2 to 8 cell diameters below its dorsal edge^41^. Ventricular cells dorsal to this bridge undergo progressive attrition, contributing to the reduction of lumen size. RG cells, spanning the entire apicobasal epithelium, were found to contact these ventricular progenitors at their apical aspect to induce their delamination away from the forming central canal. Mechanistically, RG cells were reported to secrete a soluble form of the apical polarity protein Crumbs2 that promotes loss of cell polarity and delamination of the adjacent ventricular cells expressing transmembrane Crumbs2^41^. This intriguing repetitive mechanism links cell elongation with delamination and dorsal collapse.

In the context of RP development, it is worth mentioning the formation of the glycogen body, an RP-derived structure unique to Aves that is confined to the lumbar level of the neuraxis^42–44^. There is no axonal crossing at the dorsal midline that occurs at the level of the glycogen body, whereas at axial levels, rostral and caudal to it, decussation is apparent. Thus, the glycogen body was suggested to serve as a physical barrier for axonal decussation at the sciatic plexus level. This observation is of biological significance, as it may account for the alternative pattern of hindlimb locomotor activity. This contrasts with dorsal midline crossing at brachial levels that is associated with synchronous wing movements^45^. Whether the formation of the glycogen body is preceded by an earlier stretching of RP into RG and/or by subsequent establishment of dorsal ependyma at this level has yet to be explored.

### Roof plate derivatives form in a spatially restricted pattern

Lineage tracing studies and expression of specific transcription factors suggest that dorsal spinal cord RG and ependymal cells specifically derive from the RP^21,26^. Another important message emerging from recent data is that spinal cord ependymal cells in mice and humans maintain a dorso-ventral pattern of gene expression reminiscent of their embryonic predecessors^21,25,26,46^. Specifically, the dorsal ependyma was reported to express Zic (Zic1, Zic2, Zic4, and Zic5) and Msx1 transcription factors that characterize the early dorsal NT^25,46^. These Msx1^+^ radial cells were only a subset of the dorsal ependymal population, suggesting that molecular heterogeneity is maintained in the adult dorsal spinal cord, similar to observations in the nascent embryonic RP^27^. Likewise, dorsal ependymal cells in rodents express genes encoding Bmp6 and Wnt4^46^, reminiscent of the well-documented activity of both BMP and Wnt signaling pathways during early development. Together, these data indicate that mature dorsal ependyma derives from and retains some properties of the embryonic RP. The functions of the above genes and morphogens at late stages remain to be clarified (see next section).

### The roles of Wnt signaling

Expression of Wnt1 and Wnt3a, which is prominent in the early dorsal NT^39,47,48^, is maintained in stretched RP cells. In mice, Wnt-responsive progenitors are restricted to the dorsal region of the central canal, where dorsal ependymal cells are generated. Wnt secretion by RP cells is required for the proliferation of these ependymal progenitors and affects RG organization but has no effect on RG formation. Furthermore, Wnt signaling is active in postnatal and adult ependymal cells, where it contributes to their proliferation under homeostatic conditions and also following spinal cord injury^21,26^ (Figure 2).

## Common principles acting in the dorsal neural tube over time

Available results highlight a number of reiterative motifs operating in the dorsal NT during development that are briefly summarized below. For instance, the onset of NC delamination is regulated by a dynamic counter gradient of BMP and its inhibitor Noggin, and BMP is a master regulator of NC EMT^49–51^. Likewise, it appears that one of the mechanisms responsible for the end of NC production and EMT involves an interplay between BMP signaling and at least three of its inhibitors (Grem1, HES4, and BAMBI) that are specifically upregulated in the nascent RP^5^.

Canonical Wnt signals emanate from the dorsal NT and are mitogenic for early NC cells downstream of BMP^48^, as well as for more ventrally localized neuroepithelial cells^52^, and seem to keep this activity vis-à-vis developing and adult ependymal cells^21,26^ (Figure 2). Although Wnt signaling via β-catenin plays an essential role in proliferation, cell-intrinsic and environmental properties change over time, probably modulating the nature of cellular responses. Thus, the possibility that Wnts perform additional functions at later development remains open.

The RA pathway also reveals a continuum of different activities toward dorsal NT progenitors. At gastrulation, RA is required for NC specification. Next, during somite formation, somitic RA is necessary for the onset of emigration of specified NC progenitors, but at advanced somite stages, it is dispensable for the subsequent maintenance of NC EMT^53^. Recently, we reported that RP-derived RA ends NC production by inhibiting BMP/Wnt signaling without affecting dorso-ventral patterning of the neuroepithelium^5^. Together, this highlights a dynamic and context-dependent behavior of RA at sequential stages of NC ontogeny. These results also suggest that a network linking RA activity with BMP and Wnt signaling pathways might operate throughout dorsal spinal cord development.

EMT, an evolutionarily conserved morphogenic process, is defined by the loss of epithelial characteristics, acquisition of a mesenchymal phenotype, and altered patterns of intercellular communication leading to changes in cell migration and invasion^54,55^. Data discussed here highlight reiterative events of EMT during dorsal NT ontogeny. NC EMT is a temporally regulated process that involves reciprocal interactions between NT and adjacent mesoderm to modulate the activity of BMP/Wnt factors (reviewed in 20). This raises the question of whether the mechanisms acting upstream of Crumbs2-mediated delamination of dorsal ventricular cells by RG^41^ resemble those mediating NC EMT.

## Conclusions and open questions

The preceding results indicate that RA activity at NC and RP stages, respectively, is differently interpreted by the cells. This might be accounted for by the origin of the signal stemming from either mesoderm or NT. A different origin could account for various forms of local interactions mediated through membrane protrusions^56–58^, either in addition or alternative to signal diffusion through the extracellular space. This question is still wide open to investigation.

Recent data highlight an array of molecular differences between premigratory NC and young RP cells^27^. It would be interesting to examine whether the expression of those genes that define the nascent RP is also maintained throughout RG and ependymal formation and, if so, to investigate their functions at the various stages.

Likewise, it remains unknown how these structures segregate at different levels of the neural axis. For instance, using single-cell spatial transcriptomics in embryos aged 7 somite pairs, a recent study revealed the existence of various subdomains in the dorsal NT corresponding to the midbrain level; one of these subsets was located ventral to the premigratory NC and displayed both pluripotency and neural genes^59^. This raises the intriguing possibility that stem-like cells in this early niche are the progenitors of later RP and RG. Notably, these reported “neural stem cells” do not express classic NC markers such as *Foxd3*, *Sox9*, or *Snai2*, whereas, in the trunk, RP progenitors stem from a Foxd3^+^ lineage^28^. Hence, the reported neural stem cells in Lignell *et al*.^59^ might not be the source of RP and RG; alternatively, RP and RG cells might differ in their origin along the axis.

The results discussed here also bear clinical significance. Spinal cord ependymal cells may behave as a pool of quiescent stem cells with neurogenic and/or gliogenic potential to treat spinal cord injury^60^. A future challenge will be how to harness this endogenous potential for designing regenerative strategies. Furthermore, abnormal regulation of Wnt/β-catenin signaling is at the origin of several malignant tumors^61^. Since Wnts are also key regulators of ependymal proliferation^21,26^, this raises the question of whether the formation of ependymomas and/or additional central nervous system tumors might result from abnormal levels of Wnt activity.
